# An algebra-based method for inferring gene regulatory networks

**DOI:** 10.1186/1752-0509-8-37

**Published:** 2014-03-26

**Authors:** Paola Vera-Licona, Abdul Jarrah, Luis David Garcia-Puente, John McGee, Reinhard Laubenbacher

**Affiliations:** 1 Center for Quantitative Medicine, University of Connecticut Health Center, Farmington, CT 06030-6029, USA; 2 Department of Cell Biology, University of Connecticut Health Center, Farmington, CT 06030, USA; 3 Department of Mathematics and Statistics, American University of Sharjah, Sharjah, UAE; 4 Department of Mathematics and Statistics, Sam Houston State University, Huntsville, TX 77341-2206, USA; 5 Mathematics and Statistics Department, Radford University, Radford, VA 24142, USA; 6 Jackson Laboratory for Genomic Medicine, Farmington, CT 06030, USA

**Keywords:** Reverse-engineering, network inference, Boolean networks, molecular networks, gene regulatory networks, polynomial dynamical systems, algebraic dynamic models, evolutionary computation, DNA microarray data, time series data, data noise

## Abstract

**Background:**

The inference of gene regulatory networks (GRNs) from experimental observations is at the heart of systems biology. This includes the inference of both the network topology and its dynamics. While there are many algorithms available to infer the network topology from experimental data, less emphasis has been placed on methods that infer network dynamics. Furthermore, since the network inference problem is typically underdetermined, it is essential to have the option of incorporating into the inference process, prior knowledge about the network, along with an effective description of the search space of dynamic models. Finally, it is also important to have an understanding of how a given inference method is affected by experimental and other noise in the data used.

**Results:**

This paper contains a novel inference algorithm using the algebraic framework of Boolean polynomial dynamical systems (BPDS), meeting all these requirements. The algorithm takes as input time series data, including those from network perturbations, such as knock-out mutant strains and RNAi experiments. It allows for the incorporation of prior biological knowledge while being robust to significant levels of noise in the data used for inference. It uses an evolutionary algorithm for local optimization with an encoding of the mathematical models as BPDS. The BPDS framework allows an effective representation of the search space for algebraic dynamic models that improves computational performance. The algorithm is validated with both simulated and experimental microarray expression profile data. Robustness to noise is tested using a published mathematical model of the segment polarity gene network in *Drosophila melanogaster*. Benchmarking of the algorithm is done by comparison with a spectrum of state-of-the-art network inference methods on data from the synthetic IRMA network to demonstrate that our method has good precision and recall for the network reconstruction task, while also predicting several of the dynamic patterns present in the network.

**Conclusions:**

Boolean polynomial dynamical systems provide a powerful modeling framework for the reverse engineering of gene regulatory networks, that enables a rich mathematical structure on the model search space. A C++ implementation of the method, distributed under LPGL license, is available, together with the source code, at http://www.paola-vera-licona.net/Software/EARevEng/REACT.html.

## Background

The inference, or reverse-engineering, of molecular networks from experimental data is an important problem in systems biology. Accurate methods for solving this problem have the potential to provide deeper insight into the complexity and behavior of the underlying biological systems. So far the focus has been largely on the inference of network topology, that is, on the wiring diagram representing the regulatory relationships connecting different genes [1,2].

It has been argued that one can obtain a significant improvement in performance with inference methods that make use of data that capture the dynamics of a network in response to perturbations [3]. This point of view has been adopted in the design of several recent methods that take into account time series data as well as perturbations of the network [4-7].

Making effective use of prior knowledge is also crucial in any inference problem [8], because it is usually vastly underdetermined due to insufficient data quantity and/or quality. That is, typically many different models are consistent with the available data, and prior knowledge must be used to further narrow down the space of candidate models. Prior knowledge can come in different forms such as information about the network’s sparsity or specific connectivity information. Since gene regulatory networks are known to be sparsely connected, many inference methods specify constraints to favor sparse networks in the inference process. This is achieved for example, by iteratively setting weak connections to zero [9], choosing the sparsest among all possible solutions [10], or simply imposing a maximum number of regulators per gene [11]. However, care must be taken, since the more biologically meaningful networks might not necessarily correspond to the most parsimonious solution [12]. Explicit knowledge of the network’s connectivity can be gathered from previous biological knowledge of the system in question [13-16] or from the contributed knowledge from different inference methods, particularly when heterogenous data types are available (*e.g.* steady state data *vs.* time series).

Finally, another desirable property of inference methods is the tolerance to certain levels of noise in the experimental data used. This is especially important for methods that capture dynamical properties of the network in order to avoid the problem of over-fitting the data [17]. Sources of noise include both biological and measurement noise. For methods that discretize data, such as Bayesian network or Boolean network methods [18,19], an additional source of noise comes from the necessary discretization of continuous data into categorical data.

Several inference methods have one or several of the aforementioned features. Some of these methods fall in the category of *coarse-grained models* based on discrete variables, such as Boolean networks, Bayesian networks, Petri nets, and polynomial dynamical systems [19-25]; others correspond to *fine-grained models* based on continuous variables, such as systems of ordinary differential equations, artificial neural networks, hybrid Petri nets, and regression methods [26-32] (For a broad overview of the different methods in the field, we refer the reader to [33-35]). However, there is still a need for inference methods that gather all the previously mentioned properties, and for which their mathematical frameworks can be exploited to improve the methods’ performance.

In this paper we present a novel reverse-engineering method that combines all of these relevant features. It uses input that consists of (1) time courses of experimental measurements, which can include various network perturbations, such as data from knock-out mutants and RNAi experiments, and (2) prior knowledge about the network in the form of directed edges between nodes (representing known regulatory interactions) or as information about the regulatory logic rules of individual nodes. The output of the algorithm is a family of Boolean dynamic models, from which one can extract a directed graph whose edges represent causal interactions between nodes. The Boolean dynamic models are identified by an optimization algorithm that searches through the space of Boolean dynamic models that approximate the given data and satisfy the constraints imposed by the prior biological information. An important feature of the algorithm is that it uses the expression of Boolean functions as polynomials, leading to a model search space that has a rich mathematical structure that can be exploited. This effective representation of dynamic models lends a criterion for measuring model complexity and to select models accordingly. We show that the method is robust to a significant level of noise in the data. Additionally we show that the method’s performance on the data set used in [36], compares favorably to that of several other methods. Our algorithm incorporates work contained in the first author’s Ph.D. thesis [37].

## Methods

### Modeling framework

We use the modeling framework of Boolean networks, represented as time-discrete, state-discrete dynamical systems. A Boolean network on *n* variables can be viewed as a function

$$f=(f_{1},\ldots ,f_{n}):k^{n}\to k^{n},$$

where each coordinate update function *f*_*i*_:*k*^*n*^→*k* is a Boolean function in *n* variables and *k*={0,1}.

If we use the fact that *k* supports the algebraic structure of a number system, using addition and multiplication modulo 2, then we can express each Boolean function as a polynomial function with binary coefficients, using the translation *xANDy*=*x*·*y*, *xORy*=*x*+*y*+*xy*, *NOTx* = *x*+1, while addition corresponds to the logical exclusive OR function XOR. Since *x*^2^ = *x* in *k*, each polynomial can be assumed to be square-free, that is, every variable in every term of the polynomial appears with exponent 1. With this reduction, there is in fact a one-to-one correspondence between Boolean functions in *n* variables and square-free polynomials in *n* variables over *k* (see [19]). Note that this square-free representation is equivalent to the use of Sperner systems for Boolean models’ search space reduction in [13]. We refer the reader to [38] for an overview on polynomial dynamical systems in biology and the network inference problem.

### Simulation of dynamics

Boolean models can be simulated either synchronously, by applying all coordinate functions at the same time, or asynchronously, with the coordinate functions updated sequentially, in a particular order of the nodes in the network. It is worth noting that in this mathematical framework the steady states of a dynamic model are independent of the update schedule used. However, different update schedules can result in different periodic dynamics [39-41]. While there are examples of biological systems that exhibit synchronous dynamical patterns [42,43], asynchronous simulations might be able to predict a wider range of biological behaviors. However, the exhaustive computation of asynchronous simulations becomes intractable even for moderately sized biological systems [44-46]. Thus, for the purpose of the current work, we have decided to use a synchronous update schedule for model simulation. As we will show, this assumption will also allow us to identify the Boolean functions that generate model dynamics.

### Network inference problem

The primary input to our algorithm is one or more time series of experimental measurements for all the variables associated with the nodes in the network. These can include measurements from network perturbations, such as knock-out mutants and RNAi experiments, discretized into binary data. Additional input can include prior knowledge about the network in the form of an *n*×*n* interaction matrix (*ρ*_*ij*_), where *ρ*_*ij*_ denotes the probability that a causal influence exists from node *j* to node *i*.

Given a collection of *n* variables associated with the nodes in the network, let $T^{1},\ldots ,T^{\mu_{0}}$ be the *μ*_0_ input time series. For 1≤*μ*≤*μ*_0_, $T^{\mu }:=\{t_{1}^{\mu },t_{2}^{\mu },\ldots ,t_{\alpha_{\mu }}^{\mu }\}$ represents the *μ*^*th*^ time series. Thus, $t_{j}^{\mu }$ is the *j*^*th*^ measurement in the *μ*^*th*^ time series, and consists of a vector of dimension *n*, with coordinates representing measurements for the individual *n* variables associated with the *n* nodes in the network. We assume that the data contains a proportion 0≤*ξ*<1 of noise; that is, a proportion *ξ* of entries in the time series are assumed to be “flipped” through noise. As a result of noise, or as a result of the data discretization process, the given time series might end up being inconsistent, in the sense that from a given state $t_{i}^{\mu }$ the system transitions to two different states at different points in the time courses, thus precluding fitting of the data with a deterministic model. Such inconsistencies can be eliminated by breaking the affected time courses at the points of inconsistency. That is, we eliminate all transitions from $t_{i}^{\mu }$ (rather than choosing one transition over another). We will assume for the purpose of this paper that the given input time courses are the result of removing all possible inconsistencies.

The network inference problem in our context can then be formulated as follows:

Choose a family of Boolean dynamical models { *f*:*k*^*n*^→*k*^*n*^} that: 

(1) Best fit the data, in the following sense. For each candidate *f*, generate *μ*_0_ new time series of length *α*_*μ*_ by iteratively applying *f* to the initial time points $t_{1}^{\mu }$ of the input time series. Then we require that these time series agree with the input time series, except for a fraction *ξ* of the $\left(n\times \alpha_{1})+(n\times \alpha_{2})+\cdots +(n\times \alpha_{\mu_{0}}\right)$ time series coordinate entries. To be precise, for 1≤*μ*≤*μ*_0_ and 1≤*j*<*α*_*μ*_, we search for Boolean dynamic models satisfying $f(t_{j}^{\mu })=t_{j+1}^{\mu }$ except for at most $[\;(n\times \alpha_{1})+(n\times \alpha_{2})+\cdots +(n\times \alpha_{\mu_{0}})]\times \xi$ time points in which case $f(t_{j}^{\mu })=t_{j+1}^{\mu }+1$.

(2) Conform to the prior information available about the biological system, given by the matrix (*ρ*_*ij*_).

(3) Contain Boolean coordinate functions that are as “simple” as possible, in a well-defined sense.

We emphasize that in (1), we allow models to disagree with the input data commensurate with the expected noise level. Instead, one of the optimization criteria is the Goodness-of-Fit of a given Boolean dynamic model, which measures this deviation. This relaxation is the reason for the method’s robustness to data noise, and it is one feature that sets our algorithm apart from others of this kind. We choose an evolutionary algorithm as our optimization procedure, although other optimization methods could be chosen as well.

### An efficient description of the model search space

We derive now a computationally efficient characterization of polynomial functions that fit a time series of a given length. This characterization greatly reduces the space of all polynomials that the algorithm needs to search over.

First observe that finding a dynamic model *f* = (*f*_1_,…,*f*_*n*_):*k*^*n*^→*k*^*n*^ satisfying the condition $f(t_{j}^{\mu })=t_{j+1}^{\mu }$ except for at most $[\;(n\times \alpha_{1})+(n\times \alpha_{2})+\cdots +(n\times \alpha_{\mu_{0}})]\times \;\xi$ time points, reduces to finding the individual coordinate functions *f*_*i*_:*k*^*n*^→*k* for *i* = 1,…,*n*, satisfying the condition:

(1) $$f_{i}\left(t_{j}^{\mu }\right)=t_{j+1,i}^{\mu }$$

for 1≤*μ*≤*μ*_0_, 1≤*j*<*α*_*μ*_, and 1≤*i*≤*n*, except for at most $[\;(n\times \alpha_{1})+(n\times \alpha_{2})+\cdots +(n\times \alpha_{\mu_{0}})]\times \xi$ time point coordinates.

As previously described in the *Modeling framework* Section, each function *f*_*i*_ can be expressed as a square-free polynomial in *n* variables, with coefficients in *k*. *A priori*, the search space for *f*_*i*_ is the vector space of all such polynomials in *n* variables. Since this space has dimension 2^*n*^, the number of square-free monomials in *n* variables, an exhaustive exploration of the search space quickly becomes intractable. However, each polynomial *f*_*i*_ in this space is described by the monomials that appear as summands in *f*_*i*_ (since all coefficients are either 0 or 1), and each monomial ${\text{x}}^{\text{a}}:=x_{1}^{a_{1}}x_{2}^{a_{2}}\cdots x_{n}^{a_{n}}$ is characterized by its *support*, that is, the list of variables that appear with exponent 1 in x^a^. Let supp(x^a^) denote the support of the monomial x^a^ and |supp(x^a^)| denote the number of variables in x^a^, that is, $|\text{supp}({\text{x}}^{\text{a}})|:=\sum_{i=1}^{n}a_{i}$, which we will refer to as the length of the support. We propose to reduce the search space as follows.

Note that each coordinate polynomial function *f*_*i*_ has prescribed values at exactly *m* points, where

(2) $$m:=\alpha_{1}-1+\alpha_{2}-1+\cdots +\alpha_{\mu_{0}}-1=\overset{\mu_{0}}{\underset{j=1}{\sum }}\alpha_{j}-\mu_{0}.$$

We call the integer *m* the **total length** of the input time series. Recall that the floor ⌊*r*⌋ of a real number *r* is the largest integer not greater than *r*. Then we define the set  to be the set of all monomials whose support has length at most *Φ*:=⌊log2(*m*)⌋, that is,

(3) $$ℳ:=\{x^{a}:|\text{supp}(x^{a})|\leq \Phi \}.$$

We restrict the search space to polynomials that are linear combinations of monomials in  with Boolean coefficients. As a vector space,  has dimension ∑i=0Φni. This quantity also appears in information theory as the number of bit-strings of length *n* with *Hamming weight* less than or equal to *Φ*. This expression depends on both *n* and *Φ*, thus preventing a direct comparison with 2^*n*^. In Table 1, we provide a comparison between these two quantities for the case when *Φ*=4, which corresponds to time courses of total length *m* between 16 and 31, and *Φ*=9 which corresponds to time courses of total length *m* between 512 and 1023. The rows of the table are labeled by the variables in the network.

**Table 1:** **Comparison between the dimension of**** (for different** ***Φ* values) and the dimension of the space of all Boolean functions**

**Variables**	∑i=04ni	∑i=09ni	**2**^***n***^
5	31	32	32
6	57	64	64
8	163	256	256
10	386	1023	1024
15	1941	27,824	32,768
20	6196	431,910	1,048,576
21	7547	695,860	2,097,152

The fact that each polynomial function that fits a given set of time series of total length *m* has a polynomial representation in  follows from a theoretical result in computer algebra. We present a thorough explanation of this fact in Additional file 1. However, summed up briefly, this result is based on the observation that if two different polynomials *f* and *g* have the same values at each point *t* in the input time series, that is, *f*(*t*) = *g*(*t*) for each time series point *t*, then *f* can be written as *f* = *g*+*h*, for some polynomial *h* with *h*(*t*) = 0 for each time series point *t*.

Furthermore, given a polynomial *f* and the valuations at each time series point *t*, we can find polynomials *g* and *h* with *f* = *g*+*h*, such that *h*(*t*) = 0 for each time series point *t*, and such that *g* cannot be further decomposed into the sum of two (non-trivial) polynomials *g* = *p*+*q* with *q*(*t*) = 0 for each time series point *t*. In [47], Babson *et al.* show that the *exponent vectora* = (*a*_1_,*a*_2_,…,*a*_*n*_) of any monomial **x**^**a**^ appearing in any such polynomial *g* must satisfy

$$\overset{n}{\underset{i=1}{\prod }}(a_{i}+1)\leq \mathrm{m.}$$

For a square-free monomial x^a^, this criterion translates to $2^{\left|\text{supp}({\text{x}}^{\text{a}})\right|}\leq m$, or equivalently, |supp(**x**^**a**^)|≤ log2(*m*). This justifies the definition of the set  above.

As observed in Table 1, the dimension reduction of the search space achieved in this way is significant even for reasonably large *n*. Although the search space might still be too large to admit an exhaustive exploration, this reduction makes the search space more amenable for the application of stochastic optimization algorithms. Furthermore, this reduction in the model space is not arbitrary, but is based on a careful analysis of the form of polynomials relevant for interpolating time series of a given length.

## Inference algorithm

### Inference of dynamic models as an optimization problem

We separate the binary time series $T^{1},\ldots ,T^{\mu_{o}}$ into two types: wildtype time series for *μ*=1,…,*ℓ* and, knockt-out mutant/RNAi time series for *μ*=*ℓ*,…,*μ*_*o*_. We formulate the inference problem as an optimization problem with a multi-objective function that measures: 

1) The Goodness-of-Fit of a Boolean dynamic model with respect to the input data.

2) Model complexity with respect to the *support* of the model’s coordinate polynomial functions.

3) Consistency with the network topology obtained from the dynamic model with respect to the prior knowledge of the network’s topology.

4) Consistency with any existing information about the model’s polynomial structure.

For the solution of this optimization problem we chose an evolutionary computation approach and we developed an evolutionary algorithm. Evolutionary algorithms (EAs), population-based heuristic optimization algorithms, are known to perform well on under-determined problems and noisy fitness functions [48]. Accordingly, this type of evolutionary computation approaches are suitable search methods for inferring dynamic model parameters of GRNs [49]. In particular, EAs have shown to achieve good solutions by searching a relatively small section of the entire space [50], and have been widely used in genetic data analysis and GRN inference (for an overview, see [51-53]).

Although there are many different variants of EAs, the common underlying idea behind all these methods is the same: given a population of individuals, environmental pressure causes natural selection (survival of the fittest) which causes an increase in the fitness of the population. Given a fitness function to be maximized, a population of candidate solutions is created. Based on this fitness, some of the better candidates are chosen to seed the next generation by applying recombination and/or mutation to them. Recombination or crossover is an operator applied to two or more selected candidates -the so-called parents- to form new candidates or children. Mutation applied to one candidate results in one new candidate. Executing recombination and mutation leads to a set of new candidates (the offspring) that compete, based on their fitness score, for a place in the next generation until a candidate with sufficient quality is found or a previously defined computational limit is reached [54].

In our context, polynomial dynamic models play the role of individuals in the population. Each one of these individuals are made of *n* coordinate polynomial functions. Within a given individual, polynomial functions are mutated by changing some of their monomial terms. Crossover occurs by assembling a new candidate model from optimal polynomial coordinate functions for each *i*=1,…,*n*. Additionally, to prevent a decrease of the fitness score of a given generation, some of the candidate solutions with better fitness scores are allowed to be directly cloned, that is inherited unchanged, to the next generation.

#### Multi-objective fitness function

Before describing our fitness function, it is important to observe that our optimization problem can be divided into *n* optimization sub-problems (*Divide and Conquer* Strategy): Consider a Boolean polynomial dynamic model *f* = (*f*_1_,…,*f*_*n*_) for a given generation in the EA. Based on the synchronous update schedule that we have selected for our approach, the state value of a node *i* at time *t* is computed as $x_{i}^{t}:=f_{i}(x_{1}^{t-1},\ldots ,x_{n}^{t-1})$. That is, given the coordinate function *f*_*i*_ for a node *i* to compute its state at a given time *t* for a given time series *μ*, it is enough to have the values of the time series *μ* at time *t*-1. Thus we do not require the other *n*-1 coordinate functions. Therefore, the Goodness-of-fit of a model *f* = (*f*_1_,…,*f*_*n*_) can be evaluated one coordinate function at a time. Similarly, the other optimization criteria, such as complexity, can be evaluated one coordinate function *f*_*i*_ at a time. Once each of the coordinate functions *f*_*i*_ have been evaluated, they can be assembled to a dynamic model *f*=(*f*_1_,…,*f*_*n*_) via an *n*-point crossover. This newly assembled model can then be evaluated for all the optimization criteria to estimate its fitness as a mutant or clone for the next generation in the EA.Hence, the fitness function for our EA is built as a multi-objective function consisting of the weighted sum of the different fitness criteria for each coordinate function *f*_*i*_ and the fitness of the assembled candidate dynamic models *f*=(*f*_1_,…,*f*_*n*_). We next list the different criteria.

##### Goodness-of-fit

This score measures the ability of a candidate model to fit the time series data. As previously stated, for 1≤*μ*≤*ℓ* we let $T^{\mu }=\{t_{1}^{\mu },t_{2}^{\mu },\ldots ,t_{\alpha_{\mu }}^{\mu }\}$ correspond to the wildtype input time series. Consider a candidate model *f* = (*f*_1_,…,*f*_*n*_). For each initial time point $t_{1}^{\mu }$, we consider the time series generated by iterating *f* = (*f*_1_,…,*f*_*n*_) for *α*_*μ*_ times. We compute the Hamming score as:

(4) $$H_{f}=\frac{\sum_{\mu =1}^{\ell }D_{\mu }}{n\times \left(\sum_{\mu =1}^{\ell }\alpha_{\mu }\right)},$$

where *D*_*μ*_ is the Hamming distance between input wild type time series and the input time series generated by *f*. That is, the total number of bits where the input wildtype time series and the time series generated by the candidate model *f* differ. Hence, the Goodness-of-Fit score is computed as *Model*_*FIT*_(*f*)=*W*_*HM*_(1-*H*_*f*_), where *W*_*HM*_ is the weight assigned to the model’s data fit, part of the EA parameters.

Now we consider the knock-out mutant and/or RNAi time series. For each *ℓ*+1≤*μ*≤*μ*_*o*_ we let $T^{\mu }=\{t_{1}^{\mu },t_{2}^{\mu },\ldots ,t_{\alpha_{\mu }}^{\mu }\}$ be an input knock-out mutant and/or RNAi time series, corresponding to the *r*-th gene. In this case, all the candidate models will be considered to have the *r*-th coordinate function *f*_*r*_ = 0. That is, for a given candidate model *f*=(*f*_1_,…,*f*_*n*_) we let *f*^∗^: = (*f*_1_,…,0,…,*f*_*n*_) by setting the *r*^*th*^ coordinate function *f*_*r*_ = 0, and keeping all the other coordinate functions the same as for the wildtype case. Consider the initial time points corresponding to the knockout and/or RNAi time series. From these initial time points, we consider the knockout and/or RNAi time series generated by iterating *f*^∗^ = (*f*_1_,…,0,…,*f*_*n*_) for *α*_*μ*_ times. For each *ℓ*+1≤*μ*≤*μ*_*o*_, analogously to equation 4, we compute the $H_{f^{*}}$ from comparing the knock-out and/or RNAi input time series and those generated by *f*^∗^. Similarly, we compute ${\text{Model}}_{\text{FIT}}(f^{*})=W_{\text{HM}}(1-H_{f^{*}})$.

##### Goodness-of-fit of coordinate functions

This score measures the ability of a candidate coordinate function *f*_*i*_ to fit the *i*^*th*^ column of each input time series. In the case of the wildtype time series data, we consider the time series generated by synchronously iterating the coordinate function *f*_*i*_*α*_*μ*_ times, starting at the initial time point $t_{i,1}^{\mu }$ of the *i*^*th*^ column of each input time series. We compute the Hamming score as:

(5) $$H_{f_{i}}=\frac{\sum_{\mu =1}^{\ell }D_{\mathrm{i\mu}}}{\sum_{\mu =1}^{\ell }\alpha_{\mu }},$$

where *D*_*iμ*_ is the Hamming distance between the *i*^*th*^ columns of the input wildtype time series and the time series generated by *f*_*i*_. Thus, the Goodness-of-Fit of a coordinate function *f*_*i*_ is given by ${\text{Polynomial}}_{\text{FIT}}f_{i}=W_{\text{HP}}(1-H_{f_{i}})$, where *W*_*HP*_ is the weight parameter assigned to the Goodness-of-Fit of coordinate functions. For the knock-out mutant and RNAi time series, we proceed analogously to the wildtype case (Equation 5), but considering the model *f*=(*f*_1_,…,*f*_*r*-1_,0,*f*_*r*+1_,…,*f*_*n*_), representing the knock-out mutant or RNAi experiment on the *r*^*th*^ gene (as explained in the perturbation case in the Goodness-of-Fit of candidate models.

##### Complexity score

It is important to balance the ability of a model to explain the observed data with its ability to do so simply [18]. Thus, scoring metrics with a penalty for unnecessary complexity are able to guard against over-fitting of observed data.

With the previously introduced algebraic description of the search space, we can evaluate the complexity of each coordinate function as the ratio between its total degree and *Φ* (the upper bound for the monomial support). The complexity score for a candidate model is measured as the average of the complexity scores of its coordinate functions. Notice that the complexity score in our proposed method is enabled from the algebraic identification of the upper bound for the monomial support. However other complexity criteria such as Bayesian Information Criterion [55] and Akaike’s Information Criterion [56], could be used instead.

##### Prior knowledge of network topology

Prior information about the topology of the network can be available from two different sources: (1) From previous biological knowledge and (2) from knowledge acquired from the prior use of another inference method, thus applying our method as a “meta-inference method". Prior knowledge from these two sources is encoded in the *n*×*n* matrices *BioProbMatrix* and *RevEngProbMatrix*, respectively. The entries *ρ*_*ıȷ*_ of either matrix represent the probability of a causal influence from node *ȷ* to node *ı*. For a candidate model *f*, let us consider its adjacency matrix *V*, in which an entry *a*_*ıȷ*_ is ‘1’ if *x*_*ȷ*_ appears in the *ı*^*th*^ coordinate function *f*_*ı*_ and ‘0’ otherwise. For such a matrix, consider the *ı*^*th*^ row *V*(*x*_*ı*_) = (*v*_1_,…,*v*_*ȷ*_,…,*v*_*n*_), corresponding to all variables appearing in the *ı*^*th*^ coordinate function. Let (*ρ*_*ı*1_,…,*ρ*_*ın*_) be the *ı*^*th*^ row of the *BioProbMatrix*, and let

$$\beta_{\mathrm{ıȷ}}:=\left\{\begin{array}{ll} 1-\rho_{\mathrm{ıȷ}} & \text{if}\;v_{ȷ}=0;\\ \rho_{\mathrm{ıȷ}} & \text{otherwise}. \end{array}\right)$$

Thus, the Prior Biological Knowledge score assigned to each coordinate polynomial *f*_*ı*_ is given by $\text{BioScore}(f_{ı})=\frac{1}{n}\sum_{ȷ=1}^{n}\beta_{ıȷ}$. By extension, the *BioScore* assigned to a candidate model *f* is given by

(6) $$\text{BioScore}f=W_{B}\overset{n}{\underset{ı=1}{\sum }}\frac{\text{BioScore}(f_{ı})}{n},$$

where *W*_*B*_ is the weight assigned to the model BioScore in the EA parameters.

Analogously, we compute the RevEngScore to obtain the Prior Reverse Engineering score.

#### Algorithm summary

We summarize the full algorithm as follows: **Inference of structure and dynamic polynomial models**

##### **Algorithm 1** Inference of structure and dynamic polynomial models

## Results

### Validation Part 1: assessment of robustness to data noise

Inference algorithms using a discrete modeling framework, such as Boolean or certain Bayesian methods, face an additional challenge: their performance depends on the choice of a data discretization method. Thus we separate the effect of data discretization on the method’s performance from that of robustness to data noise. We use a binary data set generated from the published Boolean model of a gene regulatory network in [40]. We added different levels of noise to the data sets to assess how robust the method is to such data noise and the effect on the dynamics prediction.

*The Segment Polarity Gene Network:* The Boolean model in [40] represents a gene regulatory network in *Drosophila melanogaster* responsible for segmentation of the fruit fly body. This Boolean model is based on the binary ON/OFF representation of mRNA and protein levels of five segment polarity genes. The authors constructed their model based on the known network topology and validated it using published gene expression data.

The expression of the segment polarity genes occurs in stripes that encircle the embryo and are captured in the Boolean model as a one-dimensional representation. Each stripe consists of 12 interconnected cells grouped into 3 parasegment primordia in which the genes are expressed in every fourth cell. The authors assumed the parasegments to be identical so that only one parasegment of four cells is considered. The variables of the dynamical system are the segment polarity genes and proteins in each of the four cells. Thus, one stripe is represented as a 15×4=60 node network which we aim to infer. In Additional file 2 we have included all the details about the Boolean model and its polynomial translation, used in this section.

#### *Input data*

Because we did not assume any prior knowledge about the amount of noise present in each one of the input data sets, we uniformly choose all EA simulations to assume 5*%* noise.

##### 

**Generation of input time series.** We generated 24 time series, including wildtype and knock-out mutant data, with a total of 202 time points (≪1*%* of the 2^21^ possible states in the system). In Additional file 2, we present a detailed description of the polynomial model used and the simulation data.

We added *ξ*=0,.01,.05 percent noise to the input data by randomly flipping *ξ*(202)(*n*) bits, respectively. Note that, since data discretization may filter out some amount of noise in the experimental data, adding noise to the already discretized data probably results in the addition of more noise than if we would have added the noise to the continuous data and then discretized it.

##### Prior knowledge about the network topology

**a) Prior Biological information.** We included only the 5 biologically obvious molecular dependencies, that is, from each one of the 5 genes in the network to their corresponding protein products.

**b) Prior use of an inference method.** Using our software as a inference method, independently of prior biological information, we input information about the network topology obtained by first applying the inference method from Jarrah *et al.* in [19] to the available data with the added noise. The method in [19] infers network topology through exact data interpolation within the polynomial dynamical systems framework.

##### Generation of parameter sets for the EA algorithm

We assess the robustness to noise under a broad sampling of parameter sets, rather than only presenting the best results after parameter tuning. To create a good sampling of multi-variable parameter sets while reducing the number of runs necessary, we used a Latin hypercube sampling (LHS) method, as introduced in [57]. From the LHS, we generated 60 different sets of parameters. In the Additional file 2, the reader can access the information on the ranges of parameter values that the LHS protocol generated.

#### *Inference of static network*

A key issue, when applying heuristic search algorithms, is their dependence on the choice of various parameters. For EA algorithms, the number of parameters that can be changed to optimize the method’s performance is often quite large. Furthermore, for inference methods that utilize EA strategies (*e.g.*[58-61]), typically a prior parameter tuning is done to evaluate overall performance based only on the set of parameters for which the EA shows the best results. We considered it important, however, to reveal a broader view of the algorithm’s performance under different choices of parameter values and identify relevant parameters to use in the evaluation of robustness to noise. As indicated before, we used an LHS method to create a broad sampling of multi-variable parameter sets while reducing the number of runs necessary.

**Figure 1:**
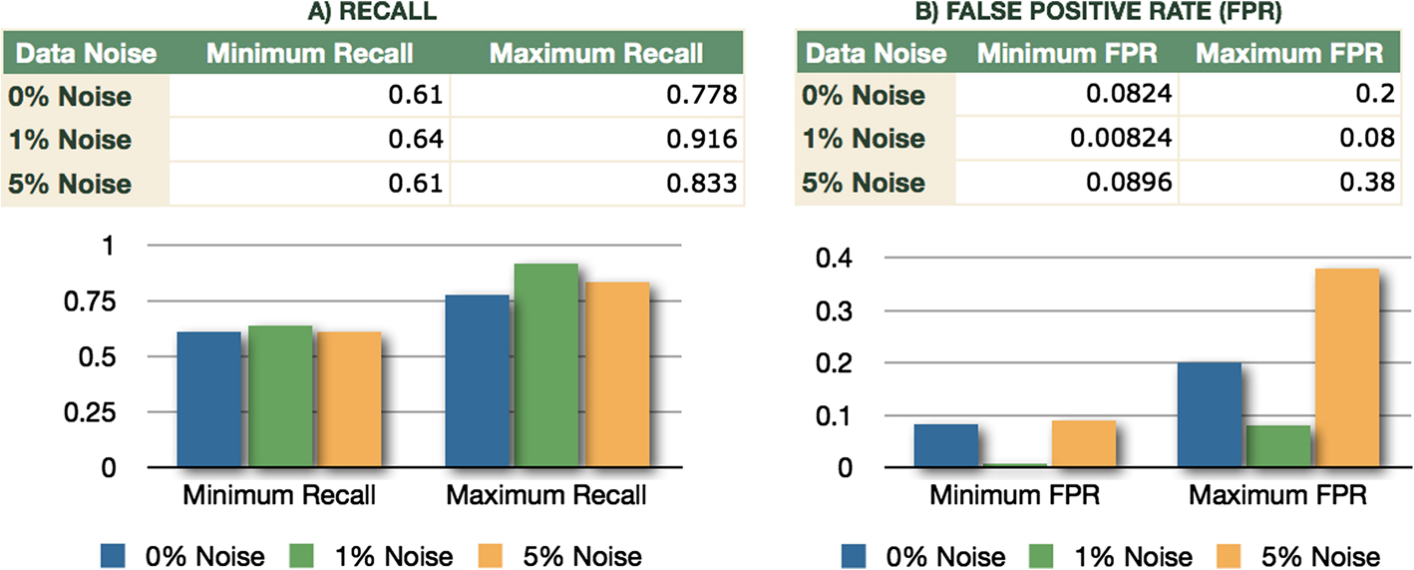
**Broad assessment of robustness under different levels of data noise.** Full range of result values of our algorithm in the presence of 0*%*,1*%* and 5*%* noise, after running triplicates of each one of the 60 sets of EA parameters. **A)** Minimum and maximum Recall values under different levels of noise. **B)** Minimum and maximum False Positive Rate (FPR) values. For our multi-parameter method, we observe that across all the ranges of values of results, the ratio between Recall/FPR on each one of the different levels of noise is always greater than 1, thus above the line of no discrimination within the Receiver Operating Characteristic (ROC) space.

**Figure 2:**
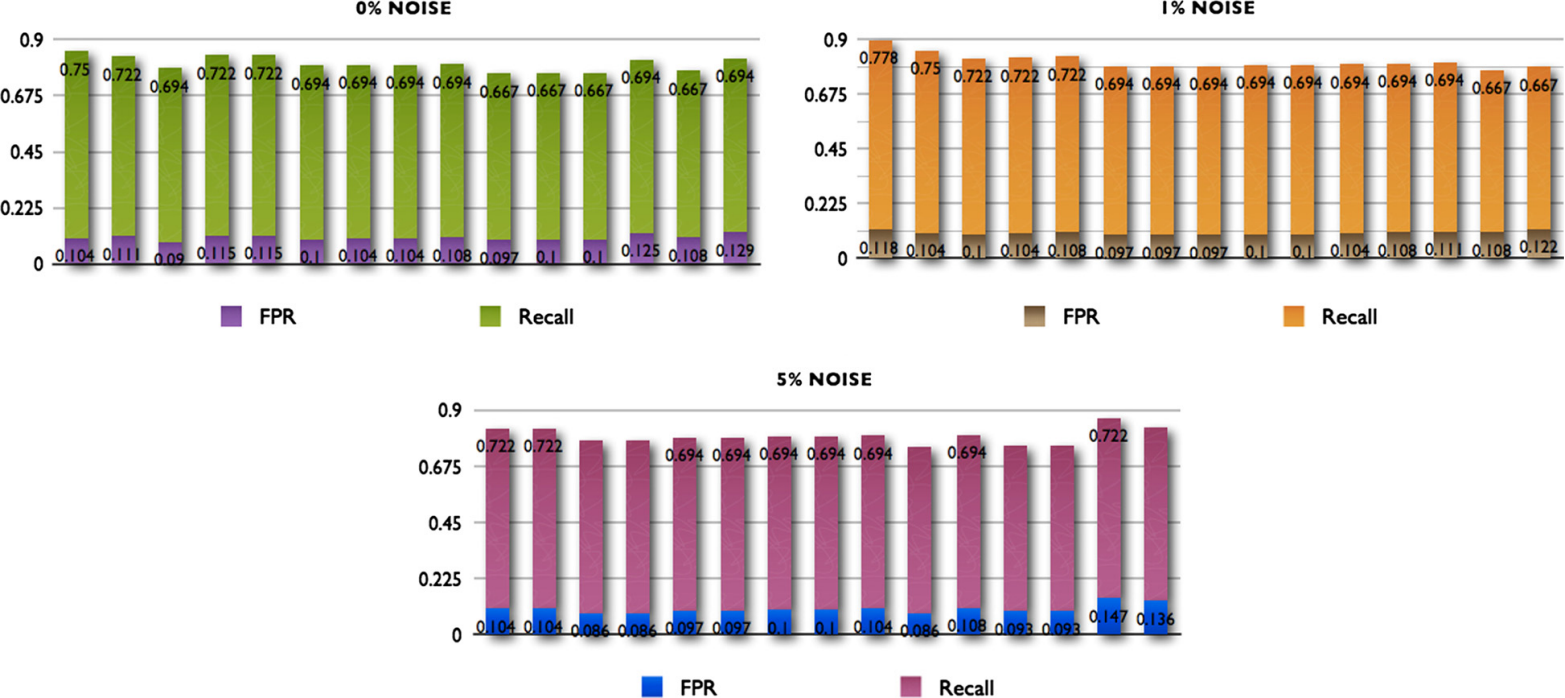
**Robustness under different levels of data noise.** Broad sample of the different pairs of Recall and False Positive Rate (FPR) values for the top ranked set of parameters. Each one of the three panels represents the stacked column graphs for the pairs of values of Recall and FPR for the three different levels of noise.

**Figure 3:**
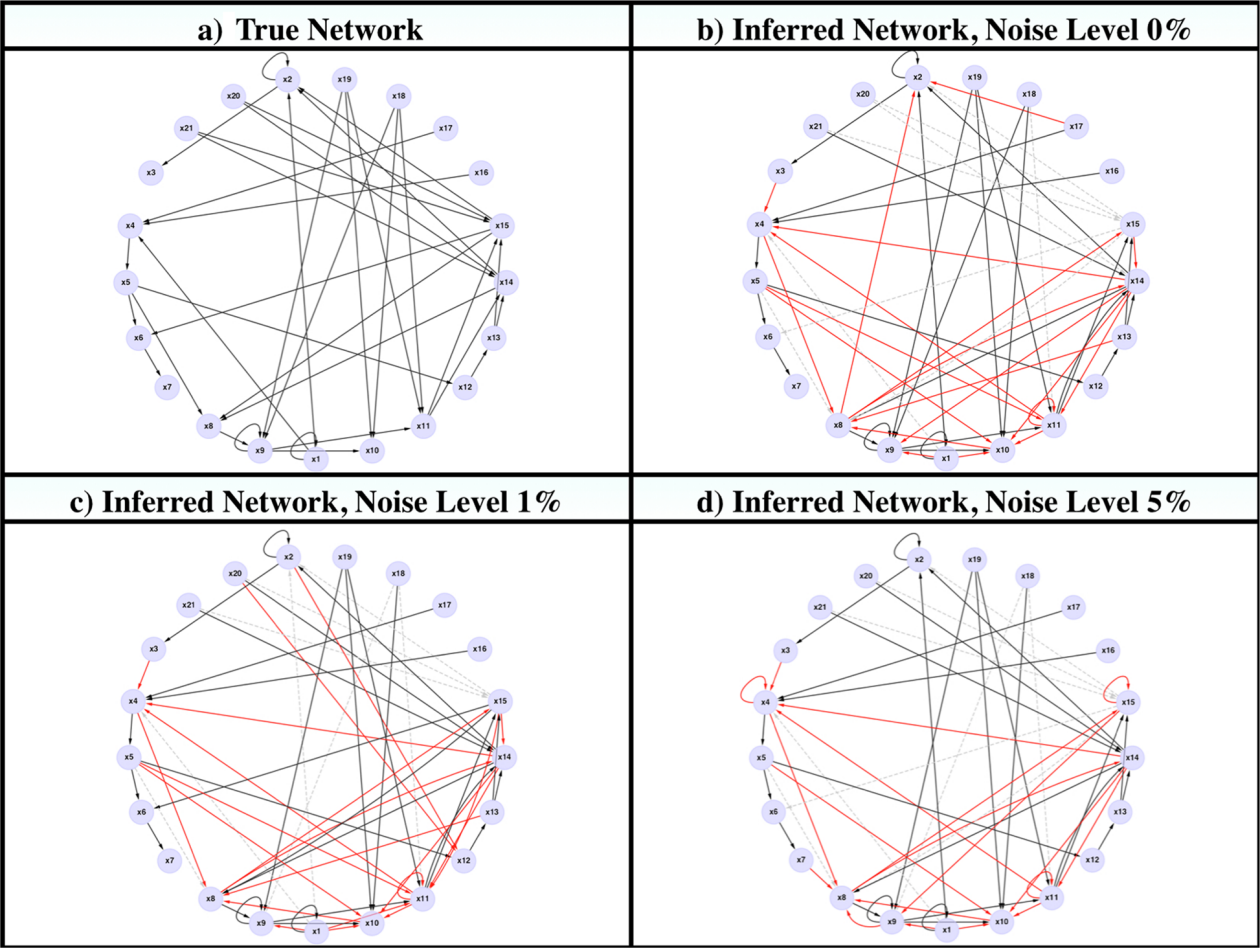
**Inferred networks topologies under different levels of data noise.** The different network topologies inferred under different levels of data noise. We show the best results obtained across the different sets of parameters from the LHS sampling method. **a)** The true segment polarity gene network; **b)** The network topology inferred under 0% of noise; **c)** The network topology inferred under 1% of noise; **d)** The network topology inferred under 5% of data noise. Solid red edges represent the inferred interactions that are not present in the real network, or that have the wrong direction (false positives), and dotted gray lines represent false negatives.

We generated triplicates for each one of the EA runs, for a total of 180 runs for each one of 3 noise levels. In Figure 1 we show the performance of our method considering all the different sets of parameters, and present the ranges -lowest to highest- of obtained values for False Positive Rate (FPR) and Recall or True Positive Rate (TPR). We observe that even for the ranges of values with the poorest performance across all the parameter sets and the different levels of noise, the ratio between FPR and Recall is always above 1; that is, according to the Receiver Operating Characteristic (ROC) space, these pairs of values fall at all times above the line of no discrimination, showing good performance of the algorithm over a wide range of parameters [62]. Additionally, we considered the parameter set that showed the highest fitness scores. In Figure 2 we show the pairs of FPR and Recall values for the three levels of noise. In Figure 3 we show a comparison of the inferred network topologies under different levels of data noise based on the best results obtained across the different 60 sets of parameters. Notice that these inferred networks are not the best solutions obtained after a tuning of parameters but simply the best results obtained from the different sets of parameters obtained by the LHS sampling method.

As mentioned earlier, we assumed no prior knowledge in terms of the amount of noise present in each one of the input data sets; thus, we uniformly choose all EA simulations to disagree with up to 5% of the data. In that sense, since for the input data set containing 0% and 1% of noise, the polynomial models are allowed to disagree with up to 5% of the bits of the input, it is not surprising that rather than being detrimental, the EA’s performance slightly improves when considering data sets with some level of noise.

#### *Inference of dynamic model*

Along with the network topology, by design, our method infers dynamic models. Thus we tested the method’s ability to predict dynamic patterns of the network across the different levels of noise.

We considered the 6 steady states retrieved in Albert and Othmer’s model [40], 3 of which correspond to patterns that have been observed experimentally. We selected the dynamic model from the output of the best performing set of parameters found in the previous section. In Table 2 we present the steady states retrieved by our method under the different levels of noise. We observe that across the different levels of noise, at least 50*%* of the expected steady states were always retrieved by the method.

**Table 2:** Steady states retrieved under the different levels of noise

**Boolean steady states**	**0** ***%* noise**	**1** ***%* noise**	**5** ***%* noise**
**retrieved from [**[40]**]**			
s1 = 0 0 0 1 1 1 1 0 0 0 1 0 0 0 0 1 1 0 0 0 0	s1	s1	s1
s2 = 0 1 1 0 0 0 0 1 1 1 1 1 1 1 0 0 0 0 1 0 1			
s3 = 0 0 0 0 0 0 0 0 1 0 0 1 1 0 1 0 0 0 0 0 0	s3	s3	s3
s4 = 0 0 0 1 1 1 1 0 0 0 1 0 0 0 0 1 0 0 0 0 0	s4	s4	s4
s5 = 0 0 0 1 1 1 1 0 0 0 1 0 0 0 0 1 0 0 1 0 1	s5		s5
s6 = 0 0 0 0 0 0 0 1 1 1 1 1 1 1 0 0 0 0 1 0 1	s6		s6

In the first column, the 6 steady states obtained after the simulation of the Boolean model in [40] for all the input initializations. In the next columns the steady states retrieved by our method under the different levels of noise are shown.

### Validation Part 2: Benchmarking with IRMA synthetic network

To test the performance of our method on expression profiles, we use the biological system in [36]. This system is a synthetic network within the yeast *Saccharomyces cerevisiae*, denoted IRMA, for “*in vivo* benchmarking of reverse-engineering and modeling approaches". The network was constructed from five genes: CBF1, GAL4, SWI5, ASH1 and GAL80. Galactose activates the GAL1-10 promoter, cloned upstream of a SWI5 mutant in the network, and is able to activate the transcription of all five network genes. The endogenous transcription factors were deleted from the organism to constrict the impact of external factors. The authors measured both time series and steady-state expression levels using two gene profiles described as Switch ON and Switch OFF, obtained by shifting the growing cells from glucose to galactose medium and from galactose to glucose medium, respectively.

#### *Input data*

##### 

**Input time series and their discretization.** The translation from continuous data to their discrete equivalent is a crucial step in preserving the variable dependencies and thus has a significant impact on the performance of network inference algorithms [63]. Although it is possible to manually chose an appropriate discretization for the data given the size of the network, we followed a systematic procedure to find the most appropriate discretization. We first compared three discretization methods, Quantile (Q), Interval (I) and SSD, a graph-theoretic method in [63], to discretize the Switch ON and Switch OFF time courses into binary states. Although none of the discretization methods was able to reproduce every observed pattern, we selected the quantile binary discretization Q2 as the method that best captures some important dynamical features in the data. In Additional file 3, we have included the comparative study of the three discretization methods with their plots.

##### 

**Prior knowledge of the network topology.** In the specific scenario of the yeast synthetic network, we observed that across the different discretization methods used, we had issues with loss of some dynamic patterns observed in the continuous data. To counteract the data limitation due to the 2-state data discretization, we used our inference method as a meta-algorithm, that is, we input a previously inferred network topology from the dynamic Bayesian method BANJO [18]. First, we performed a comparative study across different data discretization methods beyond binary, considering also 3, 4 and 5 discrete variable states. Based on BANJO’s scorings across the different data discretization methods, the highest scores are obtained when considering the quantile ternary (Q3) discretized data. In fact, selecting this more adequate discretization (according to BANJO’s scoring) improves BANJO’s performance from the results originally reported by Cantone *et al.* in [36], from [PPV = 0.30, Se = 0.25] to [PPV = 0.44, Se = 0.50] for the Switch ON time series and from [PPV = 0.60, Se = 0.38] to [PPV = 0.71, Se = 0.63] for the Switch OFF time series. Therefore, using this more optimal ternary discretization *Q*3, we input the BANJO inferred network as prior knowledge about the topology of the network.

#### *Inference of IRMA’s Static network*

##### 

**An objective benchmarking procedure.** Possible bias can occur when comparing inference methods: 1) Only methods that are “alike” to the method of interest are used for comparison purposes and/or 2) A lack of experience with the methods or software used for benchmarking, prevent an optimal use of such methods. To avoid these two biases, we decided to benchmark our method with a broad spectrum of inference methods from fundamentally different modeling frameworks (*e.g.*, continuous versus discrete modeling methods) and to exclusively use the best results reported by the authors in their corresponding publications of their own methods. With that in mind, we benchmarked our method with the broad range of inference methods proposed in [6,64,65] and [66]. They all used the IRMA network and its time series data to benchmark their methods with BANJO and TSNI [18,67], as reported by Cantone *et al*.

**Figure 4:**
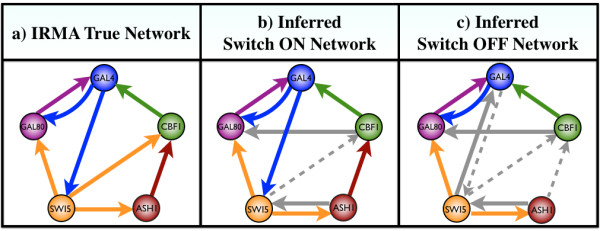
**Our method applied to experimental gene data.** The experiment measured the expression level of 5 genes after a shift from galactose-raffinose- to glucose-containing medium. **a)** The true Yeast Synthetic Network; **b)** The inferred static network from the Switch ON data set; **c)** The inferred static network from the Switch OFF data set. Solid gray edges represent inferred interactions that are not present in the real network, or that have the wrong direction (false positives), and dotted gray lines represent false negatives.

In Figure 4, we show first a comparison between the true IRMA network and the networks inferred by our algorithm for both the switch ON and OFF data. We observe for the switch ON time series data that seven edges are correctly inferred, one edge has a wrong direction, one is a false positive (CBF1 → Gal80), and only one edge is missing. As noted in [64], the Switch OFF data are a challenge due to a lack of a significant stimulus compared to the Switch ON data. In this case, our method was able to infer 5 edges correctly, one edge has the wrong direction, 2 are false positives, and 3 edges are missing.

In Table 3 we have also summarized the results obtained with our method for both IRMA’s Switch ON and Switch OFF time series and the comparison with the best results reported in each one of the aforementioned publications. We observe that in the Switch ON data, our algorithm obtains better or similar PPV in comparison with the best results across all the other methods, except for the results reported in [6], where, while having a high PPV, only 3 out of 8 edges are inferred. Additionally, for this same data set, the recall values of our method outperformed those of any of the other inference methods.

**Table 3:** Benchmarking our method’s performance with the IRMA network

	**a) Our algorithm**	**b) TD ARACNE**	**c) TSNI**	**d) Porreca** ***et al.***	**e) Zou** ***et al.***
**Switch ON network**					
PPV	0.78	0.71	0.80	1	N/A
Recall	0.88	0.67	0.50	0.63	N/A
**Switch OFF network**					
PPV	0.63	0.37	0.60	1	.8
Recall	0.63	0.60	0.38	0.63	.5

In a) the results from our algorithm; in b) the results in [64] which outperforms the standard ARACNE method reported in [36]; in c) TSNI as reported in [36]; in d) the results reported in [6] after using time series and product synthesis rates and finally, in e) the results reported in [65].

One important aspect to mention is with respect to the level of noise we assume in the data. Detailed error models have been proposed to attempt to quantify the uncertainty in gene expression data (*e.g.*, the Rosetta error model [68]) and the impact of noise on the outcome of statistical analysis of microarray data [69,70]. However, in our case, in the absence of replicate data, we could not perform such an analysis. Thus, analogous to the case of the segment polarity gene network, we selected EA simulations assuming the data sets to contain 5*%* noise.

#### *Inference of IRMA’s dynamic network model*

One of the goals of modeling gene regulatory networks is to obtain a predictive model. To assess the prediction capabilities of our method, we used the Switch ON time series as input data and we tried to predict the expression profiles in the Switch OFF experiment time series.

**Figure 5:**
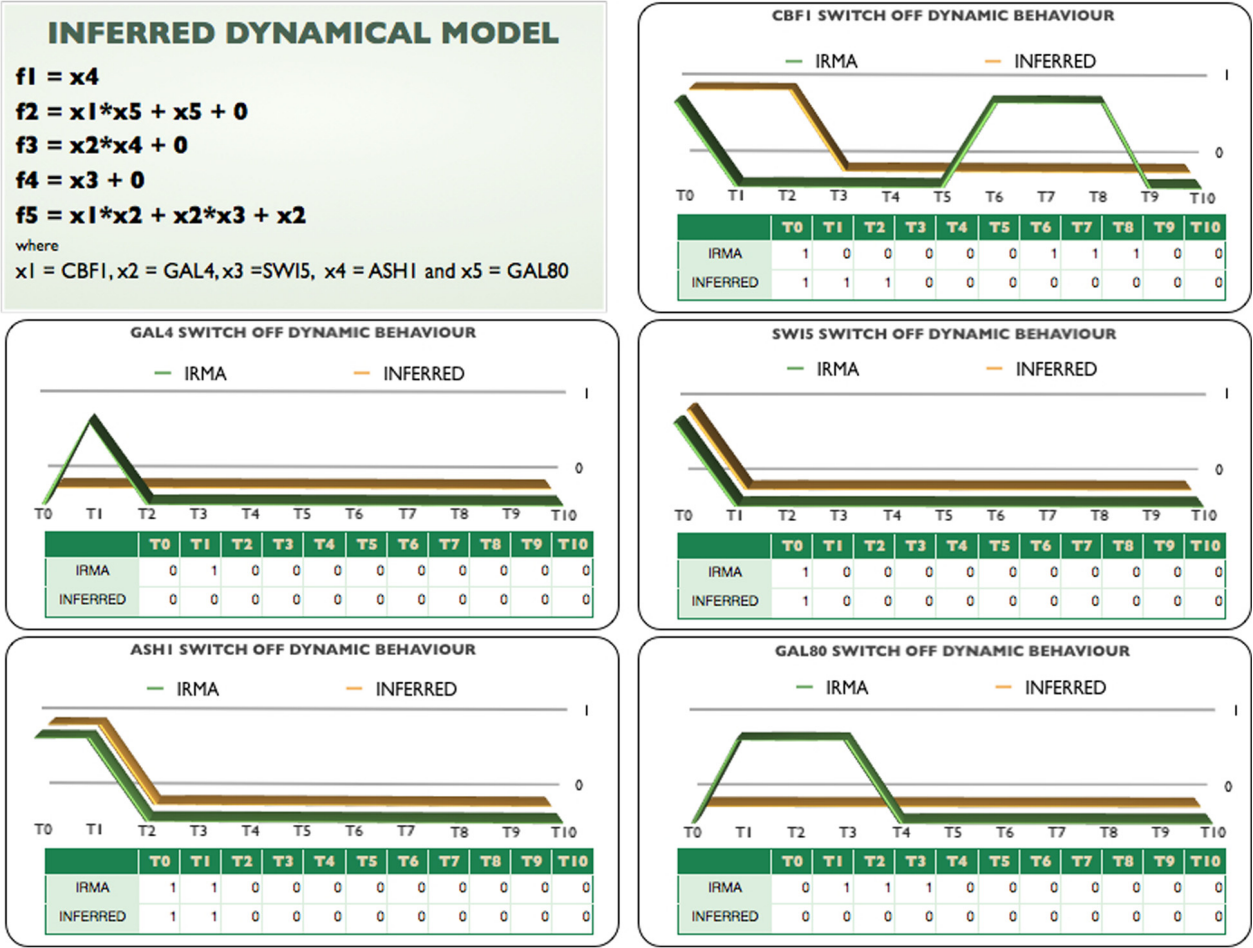
**Dynamic behavior of inferred model.** First panel shows the polynomial model inferred using the Switch ON time series. The five graphs show the Switch OFF time series behavior predicted by the inferred model, for each one of the five variables in the network. On green is shown the dynamic patterns of IRMA network. On golden is shown the dynamic patterns of the predicted dynamic model.

In Figure 5 we represent the predicted dynamic patterns of each one of the five genes for the Switch OFF data in IRMA and from our inferred dynamic model. We observe that the inferred dynamic model is capable of reproducing fully the dynamic behavior for SWI5 and ASH1. The behavior of GAL4, which is expected to switch off, shows no expression at all times in our predicted model, therefore predicting the time series except for the initial time point. Similarly, for GAL80 we observe that the expected degradation behavior of the Switch OFF time series is partly reproduced but faster than the actual IRMA time series.

Because the aforementioned last two variables are representing GAL4 and GAL80 mRNA levels when the cell’s environment is shifted from galactose to glucose medium, one would expect to observe a degradation of their mRNA levels. However, as noted by the authors in [36], the transient increase observed in the mRNA levels at the first time step of GAL4 and GAL8 is attenuated by an effect unrelated to their transcriptional regulation. This implies that the genes responsible for the regulation of such galactose-related variables will lack this dynamic information and, thus, it is natural to expect such behavior not to be predicted by any method based solely on mRNA data. The only variable that our method could not capture was CBF1.

## Discussion

### Efficient inclusion of prior knowledge

As mentioned in the introductory section, the effective use of prior knowledge is crucial to overcome the lack of quantity or quality of data. To illustrate this, consider again the IRMA network as an example. As noted in the previous section, the Switch OFF data are a challenge due to a lack of significant stimulus compared to the Switch ON data. This scenario is ideal to highlight that, with a lack of sufficient information provided by the observational data, the performance of inference methods can overcome the input data limitations by the additional consideration of prior knowledge about the topology of the network. To show this, we set up the next experiment: with the Switch OFF data as input we ran our method using as prior knowledge different amounts of information about the network topology. We created 4 networks with prior information about the network topology by considering 25*%*,50*%*,75*%* and 100% of randomly chosen true positives in the network. Our objective then is to investigate whether successively adding more prior knowledge about the network topology will also show an incremental improvement in the method’s performance. Accordingly, in Table 4, we observe the progressive improvement of our inference method after successive addition of prior information about the toplogy of the network. After the addition of just 25% of the edges of the network as a prior, our method outperforms TSNI, TD Aracne from [64] and the recall from [6]. After the addition of 75*%* of the network’s structure as prior, our method also outperforms the method described in [65].

**Table 4:** Network inference from time series data and with the successive addition of prior information about the network topology

**% Network’s topology**	**25%**	**50%**	**75%**	**100%**
**as prior input**				
PPV	0.64	0.70	0.89	0.89
Recall	0.88	0.88	1	1

We present PPV and Recall values for the assessment of our method on the IRMA’s Switch OFF network with successive addition of prior information about the network topology.

In this small example it is possible to highlight the benefit acquired from adding just partial information about the network. However, reliable sources of information are not necessarily easy to retrieve. In our method we have proposed two main sources of such prior information: 1) Prior biological knowledge of the network’s topology and, 2) Prior information about the network obtained from other inference methods applied to available data (input in our *RevEngProbMatrix*). The latter source of information is particularly useful when different types of data are available so that *ad hoc* methods can be applied to each type of data.

It is possible to imagine other scenarios in which other kinds of prior biological knowledge can be used with our method. For instance, suppose that for a given gene *i*, the maximum number of binding sites for activators/repressors is known. Then the upper bound *Φ* for the maximum monomial support of all the variables (genes/proteins) in the network can be refined for the polynomial function *f*_*i*_, describing the dynamic patterns of gene *i*. First, an unbiased initial run of the EA algorithm can be done, with the *Φ* upper bound from the input data. From these runs, one might identify the *Φ* most prevalent variables in *f*_*i*_, *i.e.*, the more likely activators/repressors of the gene in question. With these *Φ* most prevalent variables, the *i*^*th*^ row in the *BioProbMatrix* can be fixed with 1^′^*s* in the corresponding columns of these variables and the rest fixed to 0, in order to find the most optimal polynomial models. In the case that the number of most prevalent variables is less than *Φ*, several runs of the EA can be done; in each one of these runs, for the *i*^*th*^ row of the *BioProbMatrix*, one can fix to 1 the values for these variables while considering combinations of the other variables to be fixed to 1 and the rest of the variables fixed to 0, until we find best scored polynomial models.

## Conclusions

The development of algorithms for the inference of molecular networks from experimental data has received much attention in recent years, and new methods are published regularly. Most of these methods focus on the inference of the network topology and cannot use information about the temporal development of the network. Additionally, there is still a need for methods that can take different types of prior information about the network. Finally, well justified search space reductions are needed to improve the performance of inference methods.

The method we present in this paper combines several useful features: (1) it effectively uses time series data; (2) it takes into account prior information about the network; (3) it infers dynamic models so that it can predict long-term dynamic behavior of the network; (4) it is robust to noise in the input data; and, (5) it uses theoretical tools from computer algebra and a local search algorithm to efficiently explore the model search space and to optimize between model fit and model complexity.

Our method compares in general favorably with other inference methods that also utilize time series data. As we have shown here, a good strategy for increased performance is the introduction of an effective search space reduction and the combination of different inference methods.

Lastly, although our method is within the PDS modeling framework, our introduced description of the search space can be applied as well to other Boolean modeling approaches. We expect this description to be useful for Boolean methods proposed in the future or to improve the performance of existing ones.

## Competing interests

The authors declare that they have no competing interests.

## Authors’ contributions

PVL developed the design of the algorithm and helped with its encoding, performed the robustness to noise analysis and performed the benchmarking analysis. AJ participated in the design of the algorithm and supported PVL with the benchmarking analysis. LDG participated in the design of the algorithm and performed the comparison of dimensionality of the classes of Boolean functions. JM participated in the design of the inference algorithm, helped with its encoding and supported PVL in the robustness to noise analysis of the algorithm. RL participated in the design of the inference algorithm, provided funding and directed the project. All authors read, edited and approved the final manuscript.

## Supplementary Material

Additional file 1 Algebraic description of the model search space. Click here for file

Additional file 2 Segment polarity gene network. Click here for file

Additional file 3 Contains the comparison of discretization methods for the Switch ON and Switch OFF data for the IRMA network. Click here for file
