# Supplementary material for: An algebra-based method for inferring gene regulatory networks
Source: BMC Syst Biol. 2014 Mar 26;8:37. doi: 10.1186/1752-0509-8-37 (PMC4022379; doi:10.1186/1752-0509-8-37)
Supplement: Additional file 1 — Algebraic description of the model search space. [file 1752-0509-8-37-S1.pdf]

Additional file 1

Paola Vera-Licona*[1,2], Abdul Jarrah[3], Luis David Garcia-Puente[4], John McGee[5], and Reinhard Laubenbacher[1,2,6]

[1]Center for Quantitative Medicine, University of Connecticut Health Center, Farmington, CT 06030-6029, USA
[2]Department of Cell Biology, University of Connecticut Health Center, Farmington, CT 06032, USA.
[3]Department of Mathematics and Statistics, American University of Sharjah, Sharjah, UAE
[4]Department of Mathematics and Statistics, Sam Houston State University, Huntsville, TX 77341-2206 USA
[5]Mathematics and Statistics Department, Radford University, Radford, VA 24142 USA
[6]Jackson Laboratory for Genomic Medicine, Farmington, CT 06030 USA

Email: Paola Vera-Licona*- veralicona@uchc.edu; Abdul Jarrah - ajarrah@aus.edu; Luis D Garcia-Puente - lgarcia@shsu.edu; John McGee - jjmcgee@radford.edu; Reinhard Laubenbacher - laubenbacher@uchc.edu;

*Corresponding author

Algebraic Description of the Model Search Space

As stated in the main article, the reverse engineering problem of finding a Boolean network in n variables that fits a given set of time series data can be reduced mathematically to the problem of finding **coordinate polynomial functions** $f : k^n \to k$ with prescribed values at a given set of (time series) points $t_1, \ldots, t_m$ in k^n, where $k = \{0, 1\}$. We observe that in the main article we indexed each coordinate function as f_i, but here we remove the index to simplify notation, even though this may conflict with the notation used in the main paper for Boolean networks $f : k^n \to k^n$.

We also recall that $x^2 = x$ as polynomial functions in k, so any coordinate polynomial function $f : k^n \to k$ can be assumed to be square-free, that is, every variable in every term of the polynomial appears with the exponent 1. The vector space generated by all square-free monomials in n variables has dimension 2^n. In this supplementary file, we develop some polynomial algebra tools that allow us to replace this search space with a space of smaller dimension.

Each polynomial f in n variables with Boolean coefficients is completely described by the monomials that appear as summands in f. In addition, each monomial $\mathbf{x}^\mathbf{a} := x_1^{a_1} x_2^{a_2} \cdots x_n^{a_n}$ is characterized by its *support*, that is, the variables that appear with exponent 1 in the monomial. Let $\text{supp}(\mathbf{x}^\mathbf{a})$ denote the support of the monomial $\mathbf{x}^\mathbf{a}$ and $|\text{supp}(\mathbf{x}^\mathbf{a})|$ denote the number of variables in $\mathbf{x}^\mathbf{a}$, that is, $|\text{supp}(\mathbf{x}^\mathbf{a})| := \sum_{i=1}^n a_i$, which we will refer to as the length of the support.

Let $\mathcal{M}$ be the set of all monomials whose support has length at most $\Phi := \lfloor \log_2(m) \rfloor$, that is,

$$\mathcal{M} = \{\mathbf{x}^{\mathbf{a}} : |\operatorname{supp}(\mathbf{x}^{\mathbf{a}})| \leq \Phi\}, \tag{1}$$

where $\lfloor \log_2(m) \rfloor$ is the largest integer not greater than $\log_2(m)$. We restrict the search space to polynomials that are linear combinations of monomials in $\mathcal{M}$ with Boolean coefficients.

The crucial observation is that we are searching for coordinate polynomial functions $f : k^n \to k$ with specific restrictions on the values of f at each of the (time series) points $t_1, \ldots, t_m$. In other words, we are considering the set of polynomial functions $f : T \to k$, where $T = \{t_1, \ldots, t_m\}$. The set of polynomial functions on a set T is a fundamental object in the field of algebraic geometry. This set is called the *coordinate ring* of T and it is denoted by $k[T]$. We refer to [3, Chapter 5] for an elementary introduction to this topic. A fundamental result in this area states that, given a (coordinate) polynomial function $f : k^n \to k$ with specified values at $t_1, \ldots, t_m$

$$f(t_1) = b_1,\ f(t_2) = b_2,\ \ldots,\ f(t_m) = b_m,$$

where $b_1, \ldots, b_m \in k$, then any other polynomial g representing the same function $g : k^n \to k$ can be written as $g = f + h$ for some polynomial function $h : k^n \to k$ satisfying $h(t_i) = 0$, for each $1 \leq i \leq m$. This result is found in [3] where it is written in terms of *ideals (of points)* in a polynomial *ring*. Below, we present the basic ideas.

Let R denote the set of all square-free polynomials in n variables with Boolean coefficients and let $I(T) = I(t_1, \ldots, t_m)$ denote the set of all polynomials h in R satisfying $h(t_i) = 0$, for each $1 \leq i \leq m$. The set $I(T)$ is called the *ideal of points* of $T = \{t_1, \ldots, t_m\}$ and it has the following defining properties: (1) if h_1 and h_2 are polynomials in $I(T)$ the $h_1 + h_2$ is also in $I(T)$, and (2) if h is in $I(T)$ and p is an arbitrary polynomial in R, then ph is also an element of $I(T)$. Any set of polynomials satisfying properties (1) and (2) is called an *ideal* of R.

In this context, the above result stating that $g = f + h$ can be written as follows: f and g represent the same function on T if and only if $f - g$ is an element in the ideal of points $I(T)$ [3, Section 5.1, Proposition 2]. In other words, given a coordinate polynomial function $f : k^n \to k$ with specified values T, finding a coordinate polynomial function $g : k^n \to k$ with the same prescribed values on T amounts to choosing a representative of the *coset*

$$f + I(T) = \{f + h : h \in I(T)\}.$$

In our method, we select a representative from $f + I(T)$ using some type of Occam's razor principle (see [2]). We select a *minimal* element $\bar{f}$ of $f + I$ in the sense that $\bar{f}$ is an element of $f + I$ that cannot be further written as $\bar{f} = p + h$ for some non-zero polynomial $h \in I(T)$ and some polynomial $p \in R$. The selection of $\bar{f}$ is accomplished through a generalized division algorithm for multivariate polynomials.

An important property of ideals (of points) is the so-called Hilbert Basis Theorem which states that every ideal in R is *finitely generated* (see [3]). This means that there is a finite set of polynomials $H = \{h_1, h_2, \ldots, h_s\}$ in $I(T)$ with the property that

$$I(T) = \{g_1 h_1 + g_2 h_2 + \cdots + g_s h_s\},$$

where $g_1, g_2, \ldots, g_s$ are arbitrary polynomials in R. The fact that H generates $I(T)$ is denoted as $I(T) = \langle h_1, h_2, \ldots, h_s \rangle$. We observe that in each of the previous products $g_i h_i$, if a variable in any of the resulting monomials appears with an exponent greater than 1 we replace the exponent by the number 1, since $x_i^2 = x_i$ for each $i = 1, 2, \ldots, n$. The minimal element $\bar{f}$ of $f + I$ is then selected by reducing f modulo the polynomials in H via a generalized long division procedure.

In the 1-variable case, that is, when $n = 1$, this is accomplished by using the Euclidean division algorithm to find the remainder r of f under division by the unique generating polynomial h of $I(T)$. This result extends to several variables, but in this case, different orderings in the monomials of R may result in distinct remainders (in the 1 variable case there is only one sensible term order $1 < x < x^2 < \cdots$ in two variables we need to decide whether $x < y$ or $x > y$ while performing the division algorithm). In general, a *term order* $<$ is a total order on the monomials of R where the monomial 1 is the unique minimal element and

$$\mathbf{x}^{\mathbf{a}} < \mathbf{x}^{\mathbf{b}} \text{ implies } \mathbf{x}^{\mathbf{a}}\mathbf{x}^{\mathbf{c}} < \mathbf{x}^{\mathbf{b}}\mathbf{x}^{\mathbf{c}},$$

for all monomials $\mathbf{x}^{\mathbf{a}}, \mathbf{x}^{\mathbf{b}}, \mathbf{x}^{\mathbf{c}}$ in R. Given a term order $<$, every non-zero polynomial $f \in R$ has a unique *initial monomial*, denoted $\mathrm{in}_<(f)$. If I is an ideal in R, then its *initial ideal* is the monomial ideal

$$\mathrm{in}_<(I) = \langle \mathrm{in}_<(f) : f \in I \rangle.$$

The monomials which do not lie in $\mathrm{in}_<(I)$ are called *standard monomials*. The following known result is crucial in our development.

Proposition 0.1 (Proposition 1.1 in [4]) *The (images of the) standard monomials, denoted $\mathcal{B}_<$, form a vector space basis (over k) for the residue ring R/I of all cosets of the form $f + I$, with f in R.*

We further note that in our case, the residue ring $R/I(T)$ of all cosets of the form $f + I(T)$ coincides with the coordinate ring $k[T]$, this is in fact a general result. A nice consequence of the above results is that every ideal I in R has only finitely many distinct initial ideals (and hence finitely many distinct sets $\mathcal{B}_<$), see [4]. Let $\Lambda(I)$ denote the union of all distinct sets $\mathcal{B}_<$. Then any *minimal* representative of $f + I$ under any term order is a linear combination of monomials in $\Lambda(I)$. Therefore, we can reduce the model space to $\Lambda(I)$ instead of considering the space of all polynomials on n variables with coefficients in k. Unfortunately, to this date, there is no simple criterion to check if a given monomial is in $\Lambda(I)$. We will alleviate this situation by introducing a new set V_m^n with

$$\Lambda(I) \subset V_m^n \subset \text{ monomials in } R,$$

with the property that there is a simple criterion to decide if a monomial is in V_m^n.

Given a term order $<$, the set $\mathcal{B}_<$ is a staircase in $\mathbb{N}^n$. A *staircase* is a set $\lambda \subseteq \mathbb{N}^n$ such that $u \leq v \in \lambda$ (coordinatewise) implies $u \in \lambda$. The fact that the ideal $I(T)$ is the ideal of (m distinct) points $I(T) = I(t_1, \ldots, t_m)$ implies that the coordinate ring $R[T]$ is a finite dimensional vector space. Moreover, for any term ordering $<$, the cardinality of the basis $\mathcal{B}_<$ equals m, in other words, $\mathcal{B}_<$ is an m-staircase for any term order $<$; see [5]. Let V_m^n be the union of all monomials in all m-staircases in $\mathbb{N}^n$. Our arguments imply that $\Lambda(I) \subset V_m^n$. Moreover, in [6], the authors showed that V_m^n is given by

$$V_m^n = \Big\{ a \in \mathbb{N}^n : \prod_{i=1}^{n} (a_i + 1) \leq m \Big\}. \tag{2}$$

Equation (2) gives a simple criterion to check if a given monomial belongs to some m-staircase. For a square-free monomial $\mathbf{x}^{\mathbf{a}}$ this criterion translates to $2^{|\operatorname{supp}(\mathbf{x}^{\mathbf{a}})|} \leq m$, or equivalently, $|\operatorname{supp}(\mathbf{x}^{\mathbf{a}})| \leq \log_2(m)$. This justifies the definition of the set $\mathcal{M}$ in Equation 1.

References

1. Jarrah AS, Laubenbacher R, Stigler B, Stillman M: **Reverse-engineering of polynomial dynamical systems**. *Adv. in Appl. Math.* 2007, **39**(4):477–489, [http://dx.doi.org/10.1016/j.aam.2006.08.004].
2. Laubenbacher R, Stigler B: **A computational algebra approach to the reverse engineering of gene regulatory networks**. *J. Theoret. Biol.* 2004, **229**(4):523–537, [http://dx.doi.org/10.1016/j.jtbi.2004.04.037].
3. Cox D, Little J, O'Shea D: *Ideals, varieties, and algorithms*. Undergraduate Texts in Mathematics, New York: Springer, third edition 2007, [http://dx.doi.org/10.1007/978-0-387-35651-8]. [An introduction to computational algebraic geometry and commutative algebra].
4. Sturmfels B: *Gröbner bases and convex polytopes*, *Volume 8 of* University Lecture Series. Providence, RI: American Mathematical Society 1996.
5. Sturmfels B: *Solving systems of polynomial equations*, *Volume 97 of* CBMS Regional Conference Series in Mathematics. Published for the Conference Board of the Mathematical Sciences, Washington, DC 2002.
6. Babson E, Onn S, Thomas R: **The Hilbert zonotope and a polynomial time algorithm for universal Gröbner bases**. *Adv. in Appl. Math.* 2003, **30**(3):529–544, [http://dx.doi.org/10.1016/S0196-8858(02)00509-2].
