# Supplementary material for: An algebra-based method for inferring gene regulatory networks
Source: BMC Syst Biol. 2014 Mar 26;8:37. doi: 10.1186/1752-0509-8-37 (PMC4022379; doi:10.1186/1752-0509-8-37)
Supplement: Additional file 2 — Segment polarity gene network. [file 1752-0509-8-37-S2.pdf]

Additional File 2

Paola Vera-Licona*[1,2], Abdul Jarrah[3], Luis David Garcia-Puente[4], John McGee[5], and Reinhard Laubenbacher[1,2,6]

[1]Center for Quantitative Medicine, University of Connecticut Health Center, Farmington, CT 06030-6029, USA
[2]Department of Cell Biology, University of Connecticut Health Center, Farmington, CT 06032, USA.
[3]Department of Mathematics and Statistics, American University of Sharjah, Sharjah, UAE
[4]Department of Mathematics and Statistics, Sam Houston State University, Huntsville, TX 77341-2206 USA
[5]Mathematics and Statistics Department, Radford University, Radford, VA 24142 USA
[6]Jackson Laboratory for Genomic Medicine, Farmington, CT 06030 USA

Email: Paola Vera-Licona*- veralicona@uchc.edu; Abdul Jarrah - ajarrah@aus.edu; Luis D Garcia-Puente - lgarcia@shsu.edu; John McGee - jjmcgee@radford.edu; Reinhard Laubenbacher - laubenbacher@uchc.edu;

*Corresponding author

1 The Segment Polarity Gene Network

In [1], a Boolean model was proposed based on the binary ON/OFF representation of mRNA and protein levels of five segment polarity genes. The genes of this network include engrailed (en), wingless (wg), hedgehog (hh), patched (ptc), cubitus interruptus (ci) and sloppy paired (slp), coding for the corresponding proteins, represented by capital letters (EN, WG, HH, PTC, CI and SLP). Two additional proteins, resulting from transformations of the protein CI, also play important roles: CI may be converted into a transcriptional activator, CIA, or may be cleaved to form a transcriptional repressor CIR.

The expression of the segment polarity genes occurs in stripes that encircle the embryo, captured in the model rather as a one-dimensional representation that consists of a stripe of 12 interconnected cells, grouped into 3 parasegment primordia, in which the genes are expressed in every fourth cell. In Table 1 is the model by Albert and Othmer for the version used to determine steady states of the system. In Albert and Othmer [1], parasegments are assumed to be identical, so that only one parasegment of four cells is considered: the variables are the expression levels of the segment polarity genes and proteins (listed above) in each of the four cells. The authors assume the parasegments to be identical, so only one parasegment of four cells is considered: the variables are the expression levels of the segment polarity genes and proteins in each of the four cells. Hence, one stripe is represented as a $15 \times 4 = 60$ node network which we aim to infer.

In Table 2 we present the polynomial version of the subnetwork of interest, with some modification to the one introduced in [3].

1.1 Input Data Generation

1.1.1 Generation of Input Data and Data with Noise

We used the Boolean initializations for the wildtype published in [1] for the 5 genes and we generated the time courses using all the 60 Boolean functions; we treated the time courses from each cell as different state conditions of the subnetwork. We constructed knockouts from these initial conditions as well, using the value zero in the knockout gene and using the zero function as the corresponding gene function of the Boolean model. Hence, we generated a total of 20 time series, adding up to a total of 202 measurements.

1.1.2 Inclusion of Prior Biological Knowledge

- We included only the 5 obvious dependencies in the network topology, that is, from each one of the 5 genes in the network to its corresponding protein: from wg to WG, en to En, hh to HH, ptc to PTC and ci to Ci.

- Structural information about the wiring diagram: we used the algorithm from [2] to score each one of the edges in the wiring diagram, once all possible wiring diagrams that are consistent with the input time courses are considered. In Table 3, we have included the dependency matrix obtained as a prior.

Table 1: **Boolean model as proposed by Albert *et al.* [1].**

SLP	$=$	$\begin{cases} 0 & \text{if } i \in \{1,2\} \\ 1 & \text{if } i \in \{0,3\} \end{cases}$
wg_i	$=$	$(CIA_i \wedge SLP_i \wedge \neg CIR_i) \vee (wg_i \wedge (CIA_i \vee SLP_i) \wedge \neg CIR_i$
WG_i	$=$	wg_i
en_i	$=$	$(WG_{i-1} \vee WG_{i+1}) \wedge \neg SLP_i$
EN_i	$=$	en_i
hh_i	$=$	$EN_i \wedge \neg CIR_i$
HH_i	$=$	hh_i
ptc_i	$=$	$CIA_i \wedge \neg EN_i \wedge \neg CIR_i$
PTC_i	$=$	$ptc_i \vee (PTC_i \wedge \neg HH_{i-1} \wedge \neg HH_{i+1})$
PH_i	$=$	$PTC_i \wedge (HH_{i-1} \vee HH_{i+1})$
SMO_i	$=$	$\neg PTC_i \vee (HH_{i-1} \vee HH_{i+1})$
ci_i	$=$	$\neg EN$
CI_i	$=$	ci_i
CIA_i	$=$	$CI_i \wedge (SMO_i \vee hh_{i-1} \vee hh_{i+1})$
CIR_i	$=$	$CI_i \wedge \neg SMO_i \wedge \neg hh_{i-1} \wedge \neg hh_{i+1}$

Table 2: **Polynomial representation of the Boolean subnetwork of interest**

F_1	$=$	x_1
F_2	$=$	$(x_{15}+1)[x_1x_{14}+x_2(x_{14}+x_1+x_1x_{14})+x_1x_2x_{14}(x_{14}+x_1+x_1x_{14})]$
F_3	$=$	x_2
F_4	$=$	$(x_{16}+x_{17}+x_{16}x_{17})(x_1+1)$
F_5	$=$	x_4
F_6	$=$	$x_5(x_{15}+1)$
F_7	$=$	x_6
F_8	$=$	$x_{14}(x_5+1)(x_{15}+1)$
F_9	$=$	$x_8+x_9(x_{18}+1)(x_{19}+1)+x_8x_9(x_{18}+1)(x_{19}+1)$
F_{10}	$=$	$x_9(x_{18}+x_{19}+x_{18}x_{19})$
F_{11}	$=$	$[(x_9+1)+(x_9+1)x_{18}]+x_{19}+[(x_9+1)+(x_9+1)x_{18}]x_{19}$
F_{12}	$=$	(x_5+1)
F_{13}	$=$	x_{12}
F_{14}	$=$	$x_{13}[(x_{11}+x_{20}+x_{11}x_{20})+x_{21}+(x_{11}+x_{20}+x_{11}x_{20})x_{21}]$
F_{15}	$=$	$x_{13}(x_{11}+1)(x_{20}+1)(x_{21}+1)$

$F_1 = CIR_i$	$F_2 = wg_i$	$F_3 = WG_i$	$F_4 = en_i$	$F_5 = EN_i$
$F_6 = hh_i$	$F_7 = HH_i$	$F_8 = ptc_i$	$F_9 = PTC_i$	$F_{10} = PH_i$
$F_{11} = SMO_i$	$F_{12} = ci_i$	$F_{13} = CI_i$	$F_{14} = CIA_i$	$F_{15} = CIR_i$
$F_{16} = WG_{i-1}$	$F_{17} = WG_{i+1}$	$F_{18} = HH_{i-1}$	$F_{19} = HH_{i+1}$	$F_{20} = hh_{i-1}$
$F_{21} = hh_{i+1}$				

Table 3: Dependency matrix obtained as prior. Each row i represents a coordinate function F_i; each jth column from $j = 1, \dots, 21$ represents the number of times where, in the reverse engineering method of [2], there is a causal influence from x_j to the corresponding node F_i. The last column corresponds to the number of solution sets for a given variable x_i. Thus, for $i = 1, \dots, 21$, an entry a_{ij} represents the number of times that there exists a causal influence from node x_j to F_i, out of the a_{i22} possible solution sets. So, for instance, the third row corresponding to the third variable $F_3 = WG_i$ with a 1 in the second column, indicates that there is a causal influence from variable $x_2 = wg$ to $F_3 = WG_i$, the only dependency found in Jarrah's *et al.*'s method; thus we consider this dependency with a probability 1.

1	0	0	0	0	0	0	0	0	0	0	0	0	0	0	0	0	0	0	0	0	1
7	12	3	0	0	0	0	7	4	0	4	0	3	12	2	0	7	0	0	5	0	12
0	1	0	0	0	0	0	0	0	0	0	0	0	0	0	0	0	0	0	0	0	1
1	3	6	0	0	0	0	3	0	0	4	2	2	4	2	7	7	0	0	6	0	7
0	0	0	1	0	0	0	0	0	0	0	0	0	0	0	0	0	0	0	0	0	1
0	0	0	0	1	0	0	0	0	0	0	0	0	0	0	0	0	0	0	0	0	1
0	0	0	0	0	1	0	0	0	0	0	0	0	0	0	0	0	0	0	0	0	1
0	0	0	10	1	2	5	6	2	6	0	2	5	11	0	0	6	10	0	2	0	11
4	0	0	0	0	1	0	5	5	1	4	1	0	4	0	0	2	1	5	1	0	5
5	0	0	3	5	1	0	3	6	2	5	1	0	5	0	0	2	1	6	2	0	6
5	0	0	3	5	1	0	3	6	2	5	1	0	5	0	0	2	1	6	2	0	6
0	0	0	0	1	0	0	0	0	0	0	0	0	0	0	0	0	0	0	0	0	1
0	0	0	0	0	0	0	0	0	0	0	1	0	0	0	0	0	0	0	0	0	1
0	6	6	10	0	0	0	14	0	0	22	0	22	8	8	0	0	6	5	6	5	22
0	6	6	10	0	0	0	14	0	0	22	0	22	8	8	0	0	6	5	6	5	22
17	32	45	43	77	77	31	61	34	44	56	29	50	77	29	77	54	4	46	16	50	77
9	9	0	3	3	2	4	9	9	9	0	2	5	9	0	0	9	9	0	2	9	9
0	0	0	0	0	0	0	0	0	0	0	0	0	0	0	0	0	0	0	1	0	1
0	0	0	0	0	0	0	0	0	0	0	0	0	0	0	0	0	0	0	0	1	1
0	0	0	0	0	0	0	3	0	3	2	1	1	1	0	0	3	3	0	3	0	3
0	2	1	0	0	3	3	1	0	3	1	1	0	3	0	3	0	0	3	0	3	3

Table 4: Ranges of input parameter values generated by the Latin hypercube sampling(LHS) protocol

GenePoolSize	[1686,19808]
NumCandidates	[432,8470]
NumParentsToPreserve	[2,10]
MaxGenerations	[2013,4852]
StableGenerationLimit	[102,200]
MutateProbability	[.113354,.99461]
HammingPolyWeight	[0.396076,0.645279]
ComplexityWeight	[0.0730213,0.262891]
RevEngWeight	[0.0828154,0.27439]
BioProbWeight	[0.0706279,0.27439]
HammingModelWeight	[0.717499,0.898565]
PolyScoreWeight	[0.101435,0.282501]

References

1. Albert, R. and Othmer,H. (2003) The topology of the regulatory interactions predicts the expression pattern of the segment polarity genes in Drosophila melanogaster, *J. Theor. Biol.*, **223**, 1-18.
2. Jarrah,A., Laubenbacher,R., Stigler,B., Stillman,M. (2007) Reverse Engineering of Polynomial Dynamical Systems, *Advances in Applied Mathematics*, **39(4)**, 477-489.
3. Laubenbacher,R. and Stigler,B. (2004) A computational algebra approach to the reverse-engineering of gene regulatory networks , *J. Theor. Biol.* **229**, 523-537.
